# Postanaerobic Digestion Manure Fibers as a Renewable Source of Lignin-Derived Aromatic Compounds for Microbial Upgrading

**DOI:** 10.1021/acsestengg.6c00440

**Published:** 2026-09-09

**Authors:** Brayan D. Riascos, Bumkyu Kim, Steven D. Karlen, Jason Coplien, Dennis V. Haak, Sarada Sripada, Canan Sener, Timothy J. Donohue, K.G. Karthikeyan, Daniel R. Noguera

**Affiliations:** † Department of Civil and Environmental Engineering, 5228 University of Wisconsin-Madison, 1415 Engineering Drive, Madison, Wisconsin 53706, United States; ‡ Wisconsin Energy Institute, University of Wisconsin-Madison, 1512 University Avenue, Madison, Wisconsin 53726, United States; § Great Lakes Bioenergy Research Center, 1512 University Avenue, Madison, Wisconsin 53726, United States; ∥ Department of Biological Systems Engineering, University of Wisconsin-Madison, 460 Henry Mall, Madison, Wisconsin 53706, United States; ⊥ Department of Bacteriology, University of Wisconsin-Madison, 1550 Linden Drive, Madison, Wisconsin 53706, United States

**Keywords:** manure fibers, anaerobic digestion, lignin, fed-batch, 2-pyrone-4, 6-dicarboxylic acid, microbial conversion, dicarboxylic acid

## Abstract

Postanaerobic digestion manure fibers are an abundant solid residue that remains underutilized. The substantial lignocellulosic content of digested manure opens the possibility of using it as a renewable resource for producing valuable products. In this study, we performed compositional and nuclear magnetic resonance analyses to determine lignin content and aromatic composition of these fibers. Additionally, alkaline-based treatment and reductive catalytic fractionation were evaluated as two different processes to produce aromatic-rich streams. The liquor from the alkaline treatment was subsequently utilized for microbial upgrading to produce 2-pyrone-4,6-dicarboxylic acid (PDC). The results showed that lignin in manure fibers represents 30% of the biomass (dry weight basis), with a ∼26% abundance of β-ether-linked aromatics and ∼13% *p*-coumarate moieties, indicating that the amount of lignin in the postanaerobic biomass is comparable to a moderately pretreated plant biomass. The alkaline treatment released 5.6 g aromatics/kg fibers, while reductive catalytic fractionation produced 14.0 g aromatics/kg fibers. A bioreactor operated in fed-batch mode produced a yield of ∼1 mol of PDC/mol measured aromatics at a rate of 0.2 g PDC/L·h and a titer of 2.6 g PDC/L. This study demonstrates how postanaerobic digestion manure fibers could be used in a circular bioeconomy by harnessing their lignocellulosic content to extract aromatic compounds and use them for obtaining valuable chemicals via microbial upgrading.

## Graphical abstract

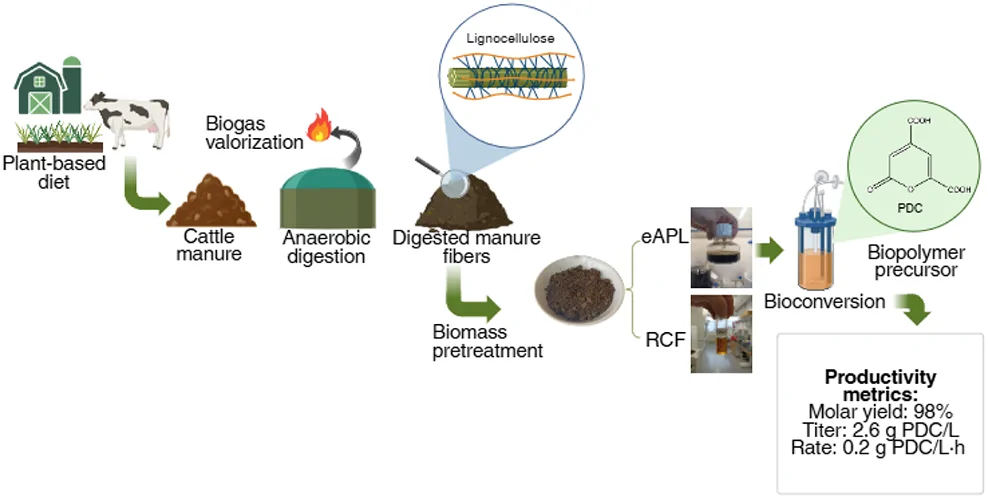

## Introduction

Approximately 3.4 billion metric tons of cattle manure are produced annually worldwide.^1^ The United States is the largest producer of beef and the third largest producer of dairy, generating 24 million metric tons and 19 million metric tons of manure annually from these activities, respectively.^2^ Cattle manure is a nutrient-rich organic residue whose management is performed through diverse disposal or treatment techniques such as land application, composting, lagoon systems, solid–liquid separation, thermochemical treatments, and anaerobic digestion.^3–5^ Anaerobic digestion of manure is implemented to gain value from the organic waste through biogas production while also providing environmental benefits related to nutrient management.^6–8^ The residue that remains after anaerobic digestion is called digestate, and it can be separated into liquid and solid fractions or directly spread on land.^4^ After the digestion process, most of the nutrients (nitrogen and phosphorus) present in manure end up in the liquid fraction of the digestate, making this fraction valuable as an organic fertilizer that is returned to agricultural soils. The recalcitrant solids that remain after the liquid fraction is recovered require additional management and proper disposal.^4,9^ A fraction of this solid digestate is used for animal bedding, although the possibility of high bacterial counts may limit this application in some regions.^10,11^ A variety of other uses have been proposed to add value to the excess digestate, including composting for use as a soil amendment, as a replacement for peat moss in containerized plant production systems for horticulture and domestic gardening, as a component of composite materials, or for biochar production.^11^

Recent research has focused on the use of microbial catalysts to generate valuable chemicals from the solid digestate.^12,13^ In this study, we hypothesized that the residual lignin in postanaerobic digestion manure fibers represents a substantial source of aromatics and that aromatics extracted from the fibers could be utilized for microbial production of dicarboxylic acids using procedures developed to valorize aromatics from energy crops.^14–16^ For instance, phenolics derived from poplar, maple, sorghum, and switchgrass have been converted into 2-pyrone-4,6-dicarboxylic acid (PDC) with engineered strains of *Novosphingobium aromaticivorans*,^17–19^ and phenolics derived from corn stover and poplar have been used to produce *cis,cis*-muconic acid with engineered strains of *Pseudomonas putida* ^20^ and *N. aromaticivorans*,^21^ respectively. Each of these dicarboxylic acids is a promising biorefining product because of its potential use as a platform chemical in polyester, nylon, and other plastic industries.^22,23^ Other products that can be obtained from lignin-derived phenolics are polyhydroxyalkanoates (PHAs), carotenoids, paracetamol, and pharmaceutical precursors such as gallic acid, pyrogallol, 3,4-dihydroxyphenyl-l-alanine, *p*-aminophenol, and acetaminophen.^15,24,25^

Lignin is a complex amorphous aromatic polymer built of phenylpropane units with *p*-hydroxycoumaryl (H), guaiacyl (G), and syringyl (S) alcohol precursors. It is the most recalcitrant polymer in lignocellulose, and its composition depends on the plant type.^15,26–28^ The composition of lignin in postdigestion anaerobic fibers has not been reported, but it may reflect the animal diet and the extent of microbial degradation within the anaerobic digester. The cattle diet encompasses a variety of feed and nutritive supplements that are necessary for beef and milk production. In general, the dairy cow diet is composed of herbaceous and leguminous plants with a high content of fiber and protein, such as warm- and cool-season grasses, corn (grain, silage, and stover), and alfalfa.^29^ Therefore, the expected lignin composition from postanaerobic digestion manure fibers can be associated with the typical composition of lignin in herbaceous plants, which comprises H-, G-, and S-type monolignols as well as hydroxycinnamates, such as *p*-coumarates and ferulates, that are mostly ester-linked to cell wall polymers.^30^ The lignocellulosic biomass present in forage fiber is incompletely degraded in the rumen or in the anaerobic digester due to its resistance to mechanical, chemical, and microbial deconstruction. Moreover, it is reported that about 50% of organic substrate in dairy cow manure is converted into biogas during anaerobic digestion,^31^ leaving a substantial refractory fraction remaining in postdigestion solid residues.

Here we describe a characterization of the lignin fraction that remains in postanaerobic digestion manure fibers using compositional and nuclear magnetic resonance (NMR) analyses. Furthermore, we evaluate alkaline treatment and reductive catalytic fractionation (RCF) as methods to produce phenolic-rich streams from these fibers and demonstrate the potential of using the extracted phenolics to produce PDC using an engineered strain of *N. aromaticivorans*.^17^ This study provides the first evaluation of using abundantly available postanaerobic digestion manure fibers to produce valuable chemicals via chemical extraction and microbial upgrading.

## Results and Discussion

### Compositional Analysis of Postanaerobic Digestion Manure Fibers

To determine the major components of postanaerobic digestion manure fibers, a compositional analysis was performed (Table 1; Table S1). This analysis shows that lignin is the major component of the fibers, followed by ash and protein. The lignocellulosic polymers lignin, cellulose, and hemicellulose constituted 30.9 ± 0.8%, 17.6 ± 0.7%, and 4.2 ± 1.2%, respectively, adding up to approximately 53% of the total mass in the postdigestion fibers on a dry basis. For comparison, compositional analyses reported in the literature for anaerobically digested manure fibers and raw manure are included in Table 1.

**Table 1:** Lignocellulose Content of Digested Manure Fibers and Other Related Biomass

		*Biomass composition (% dry matter)*				
*Biomass*	*Cellulose*	*Hemicellulose*	*Lignin*	*Crude protein*	*Ash*	*Reference*
Digested manure fibers	17.6 ± 0.7	4.2 ± 1.2	30.9 ± 0.8	21.8 ± 0.2	22.6 ± 0.4	This study
	23.6 ± 0.3	17.5 ± 0.5	27.6 ± 0.1	nr	13.7 ± 0.1	Elumalai et al.^32^
	14.3 ± 0.9	9.5 ± 0.4	42.6 ± 1.2	nr	nr	Khan and Ahring^33^
Raw manure^a^	22	17	11	23	11	Ingle et al.^13^
	24.2	20.9	13.2	14.8	20.7	Rico et al.^9^
	27.4	12.2	13.0	18.1	16.2	Chen et al.^34^
	21.89 ± 0.38	12.47 ± 0.32	13.91 ± 0.45	16.44 ± 0.53	nr	Liao et al.^35^

a Standard deviations are provided if they were reported in the noted reference; nr = not reported.

Notable in this comparison (Table 1) is the higher content of lignin in the digested manure fibers (ranging from 28% to 43%) compared with the lignin content of raw manure (ranging from 11% to 14%). Compared with the lignin content of lignocellulosic feedstocks that have been studied for microbial funneling of extracted aromatics, the postdigestion fibers have a higher lignin content. For instance, Perez et al.^18^ reported the lignin content of maple, poplar, sorghum, and switchgrass as ranging from 16% to 25%. In addition, the somewhat lower cellulose content in digested manure fibers (ranging from 14% to 24%) compared with that reported for raw manure (ranging from 22% to 27%), and to a lesser extent the hemicellulose content (ranging from 4% to 18% in digested manure fibers and 12% to 21% in raw manure), may suggest that some biodegradation of cellulose and hemicellulose polymers takes place in the anaerobic digestion environment, whereas lignin remains the most recalcitrant of the three polymers. This is consistent with NMR-based studies that characterize the degradation of organic matter during anaerobic digestion, which show that the structural carbohydrates (i.e., cellulose and hemicellulose) are more easily hydrolyzed than the aromatic components in lignin.^36–38^ This also agrees with existing literature on the biodegradation of plant fiber during animal digestion and the breakdown of manure in anaerobic environments.^39–42^ The diverse community of anaerobic bacteria and fungi in cow rumen and in anaerobic digesters can hydrolyze and solubilize structural carbohydrates into simple sugars that are further converted into volatile fatty acids and, subsequently, to methane.^40^ In contrast, the lignin macropolymer remains as a refractory component that is less susceptible to anaerobic cleavage,^39^ although published data suggest that there is some anaerobic microbial activity on the lignin polymer.^26,28,43^

In anaerobic digestion, the low degradability of lignin is also considered to limit the breakdown of other components. Indeed, several studies have reported the disruptive influence of lignin on anaerobic digestion of dairy manure, attributing low biogas yields to the role of lignin in blocking accessibility to hydrolyzable carbohydrates in cellulosic fibers.^12,44–46^ Thus, the higher lignin content observed in digested manure fibers compared with raw manure (Table 1) agrees with expectations that lignin is more recalcitrant than carbohydrate-based polymers. Table 1 also exemplifies the large variability in the composition of raw manure and digested manure fibers reported in the literature. Such variability could be attributed to many factors, including animal diet, digester temperature, and digester retention time, among others. It is also possible that variability may result from differences in the analytical methods used across studies.

### NMR Analysis of Lignin in Anaerobically Digested Manure Fibers

An NMR ^13^C/^1^H–HSQC analysis was performed on the lignin that was extracted from the postanaerobic digestion manure fibers (Figure 1). Structural units identified from different cross-peaks include aromatic protons of *p*-hydroxyphenyl units, represented in the figure as H_2/6_ (the H_3/5_ signal overlaps with G_5/6_ signals), syringyl units represented as S_2/6_, and guaiacyl units represented as G_2_ and G_5/6_ labeled peaks. In addition, the presence of *p*-coumarates in the fibers was evident from several elements in the NMR spectrum (*p*CA_8_, *p*CA_2/6_, *p*CA_7_ and *p*CA_3/5_), indicating the presence of *p*-coumarate moieties in the lignin. Furthermore, methoxyl groups were observed as well as signals corresponding to aromatic β-aryl ethers A_γ_, A_α_, and A_βS_ in the region δH ∼3.3–4.5 ppm and δC ∼59–63 ppm, indicating the presence of side chains and residual ether-linked lignin. The integration of these signals yielded an estimate of the syringyl-to-guaiacyl (S/G) ratio of 0.72 (S:G 42:58). Using a monolignol composition-adjusted methoxy signal as a 100-unit basis, 26% of the interunit linkages are β-O-4 ethers, and 13.4% of the monolignols have *p*-coumarate esters.

**Figure 1:**
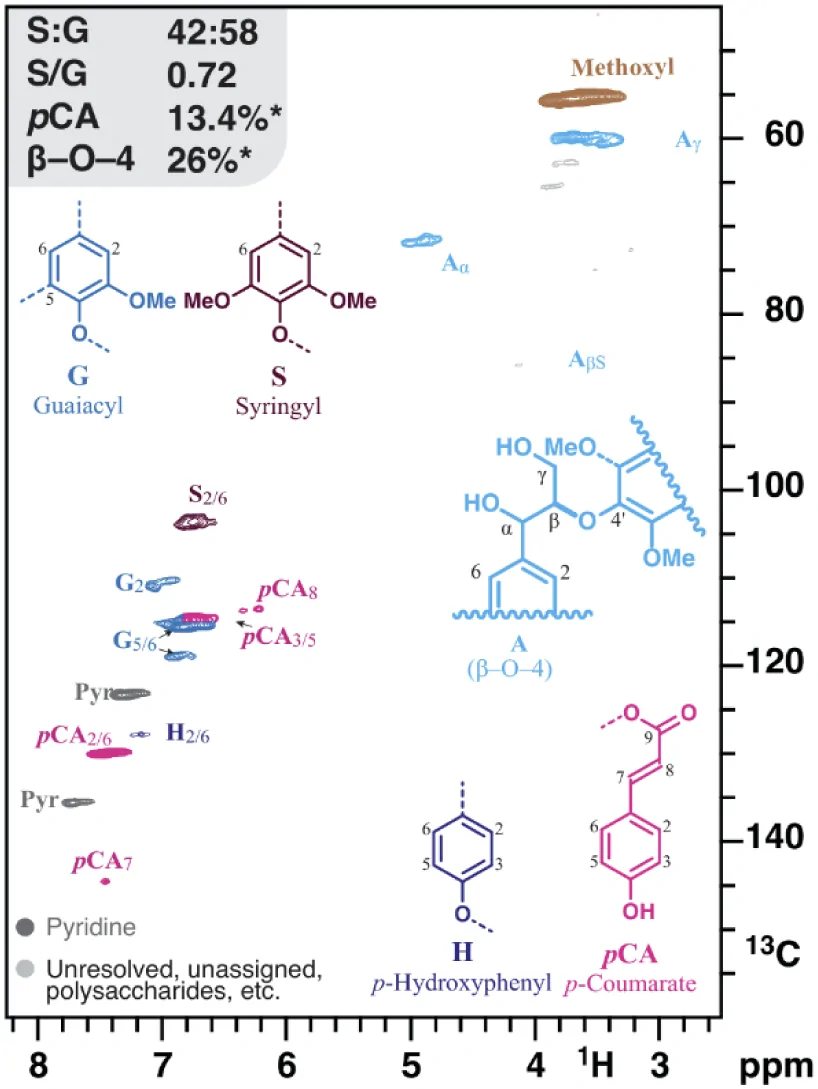
NMR spectroscopy ^13^C/^1^H–heteronuclear single quantum correlation (HSQC) of digested manure fibers. The asterisk (*) in *p*CA and β-O-4 denotes that the signals were normalized to the monolignol content as calculated by the weighted composition of the methoxy signal (1 methoxy for G and 2 methoxy for S).

In general, β-aryl ether bonds comprise 40% to 80% of the lignin interunit linkages in grasses.^27,47–50^ The estimated abundance of β-O-4 linkages in the digested manure fibers was only 26%, which is less than that predicted to be present in forage. The apparent reduction in β-O-4 linkages under anaerobic conditions is consistent with recent NMR-based evidence that the anaerobic fungi *Neocallimastix californiae*, isolated from a ruminant gut microbiome,^51^ can break this chemical bond in grasses and hardwood lignin.^43^ Thus, we hypothesize that some lignin degradation may have occurred under anaerobic conditions, either in the rumen, during manure storage, or in the anaerobic digester. Although cleavage of interunit linkages in lignin is typically mediated by oxygen-dependent ligninolytic enzymes such as laccases and peroxidases,^52^ these enzymes are not expected to be abundant or active in anaerobic environments.^26,28^ However, lignin breakdown under anaerobic conditions has been reported to occur,^28^ and information about lignocellulosic active enzymes is starting to emerge.^43^ Thus, although the contributions of anaerobic organisms to lignin degradation in anaerobic environments are not fully understood, the low β-O-4 linkage content of the digested manure fibers suggests that partial lignin degradation may have occurred in these anaerobic environments. Further investigation of the variations in lignin composition from raw manure to postdigestion fibers may provide additional insights into the potential for anaerobic cleavage of the β-O-4 linkage in these environments.

Another important difference between the NMR spectrum of postdigestion manure fibers and NMR spectra of lignin from grasses typically used as dairy cow feed^50^ is the absence of signals for ferulic acid and its conjugates, which are common pendant aromatics in grasses. Anaerobic release of ferulic acid from arabino-ferulate-containing hemicellulose and hemicellulose-lignin complexes has been shown to be mediated by esterases produced by anaerobic fungi.^53–55^ Thus, we hypothesize that such activity could result in the apparent reduction in ferulate signals compared to what is found in grasses.

### Production of Aromatic-Rich Solutions from Anaerobically Digested Manure Fibers

Based on the findings of high lignin content, we investigated chemical methods to extract aromatic compounds from the digested manure fibers. The presence of *p*-coumarates in these fibers prompted us to investigate treatment methods that typically cleave these pendant groups from grasses, such as alkaline pretreatment.^19,56^ Furthermore, the detection of β-O-4 linkages by NMR indicated the presence of oligomeric lignin in the fibers, and thus, RCF was also evaluated since this method is known to extract lignin, cleave β-O-4 linkages, and, when a Pd/C catalyst is used, produce low-molecular-weight G- and S-propanol phenolics and hydrogenated methyl esters of phenolic compounds that are compatible with downstream microbial funneling to desired chemicals.^18^ Moreover, we selected these two methods because they have been previously used for PDC production with *N. aromaticivorans*.^16–19,57,58^

For extraction and concentration of monoaromatics from the digested manure fibers, a modification of alkaline pretreatment that involves a sequence of alkaline treatment, acid digestion, and liquid–liquid extraction to generate an extracted alkaline pretreatment liquor (eAPL) was implemented.^16^ LC-MS analysis of the resulting eAPL (Table 2, Figure S1) revealed that the total concentration of phenolics in the solution was ∼55 mM, with *p*-coumaric acid (∼46 mM) representing 84% of the total aromatics extracted. Other aromatics were extracted at concentrations lower than 5 mM each, including ferulic acid, syringic acid, vanillic acid, 3-methylgallic acid, *p*-hydroxybenzoic acid, and protocatechuic acid (Table 2). These extractions produced an aromatic yield of 5.6 g phenolics/kg dry fibers (see Figure S2 for aromatic yields on a kg lignin basis). A similar extraction method to recover phenolics from poplar biomass yielded 11 g phenolics/kg dry poplar.^16^ Compared to yields obtained from corn stover (9.2 g phenolics/kg dry corn stover) and sorghum (9.0 g phenolics/kg dry sorghum) using alkaline pretreatment methods,^19,56^ the yield from digested manure fibers is ∼38% lower, reflecting a loss of pendant phenolics typically recoverable from biomass. It is possible that future improvements in the extraction process could increase phenolic yields.

**Table 2:**
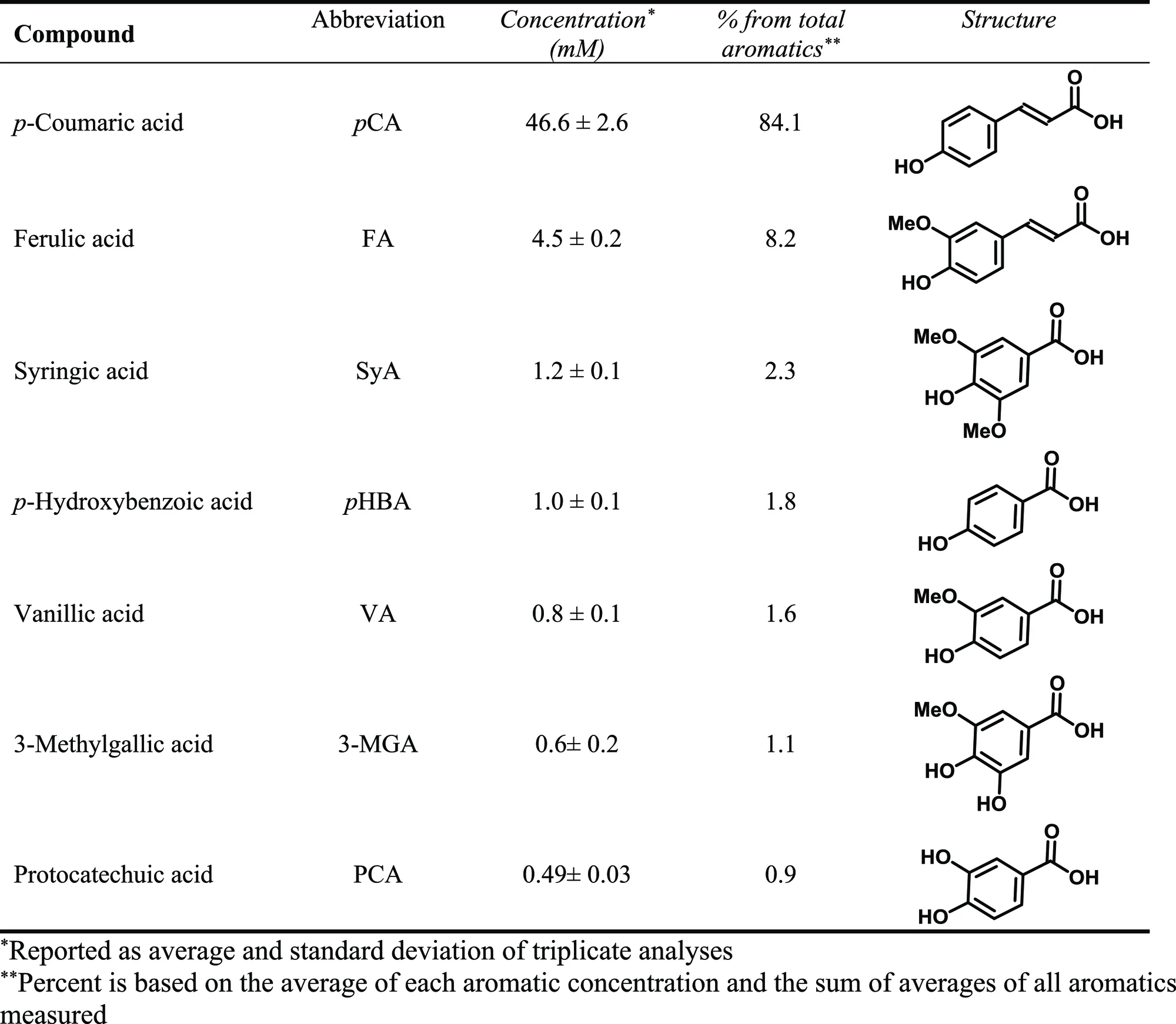
Aromatic Compounds Quantified in eAPL from Postanaerobic Digestion Manure Fibers and Standard Deviation of Triplicate Analyses

Many grasses have a ratio of *p*-coumaric to ferulic acids close to 1:1^50^ and forage plants such as alfalfa have relatively similar ferulic acid and *p*-coumaric acid content,^59^ whereas the eAPL solution had a *p*-coumaric acid to ferulic acid ratio of 10:1 (Table 2) and ferulate signals were not detected in the NMR spectrum (Figure 1). Thus, our results indicate that the linkages of ferulic acids to the biomass fiber are likely to be more susceptible to cleavage in anaerobic digestion environments than those involving *p*-coumaric acid. This could be explained by the fact that ferulic acid acylates the noncellulosic polysaccharides, glucuronoarabinoxylans, in greater abundance than *p*-coumaric acid.^60–62^ In contrast, *p*-coumaric acid acylates the γ-position in the phenylpropane side chains in grasses at a greater amount than ferulic acid, both attached primarily to syringyl units.^60,63,64^ Therefore, if ferulic acid is attached to structural sugars, it is possible that the cleavage of the arabinoxylan bond has been performed by microbial consortia in both the cow rumen and the anaerobic digester, causing arabino-ferulate esters to be removed from the carbohydrate polymer.^53–55^ Consequently, once the digestate is taken out of the digester and is separated into its solid and liquid fractions, any arabino ferulates that have not been anaerobically degraded may end up in the liquid fraction and not be retained in the solid fraction of the digestate, thus unavailable for extraction from the remaining manure fibers, explaining the low concentration of ferulic acid in the eAPL (Table 2) and the reduction in ferulate signals in the NMR spectra (Figure 1).

RCF was also evaluated as a deconstruction method that breaks interunit linkages in lignin, potentially facilitating a greater release of phenolics from the digested manure fibers than those extracted through the alkaline-based process. In RCF, the lignocellulosic biomass is fractionated, lignin is dissolved out of the polysaccharide matrix by the solvent (e.g., methanol), and then interunit linkages in lignin are catalytically cleaved.^18,65^ The monoaromatics recovered from the postanaerobic digestion manure fibers with RCF, with methanol as the solvent and Pd/C as the catalyst, included H- and G-ethyl phenolics, G- and S-propyl phenolics, and hydrogenated methyl esters of *p*-coumarate and ferulate (Figure 2).

**Figure 2:**
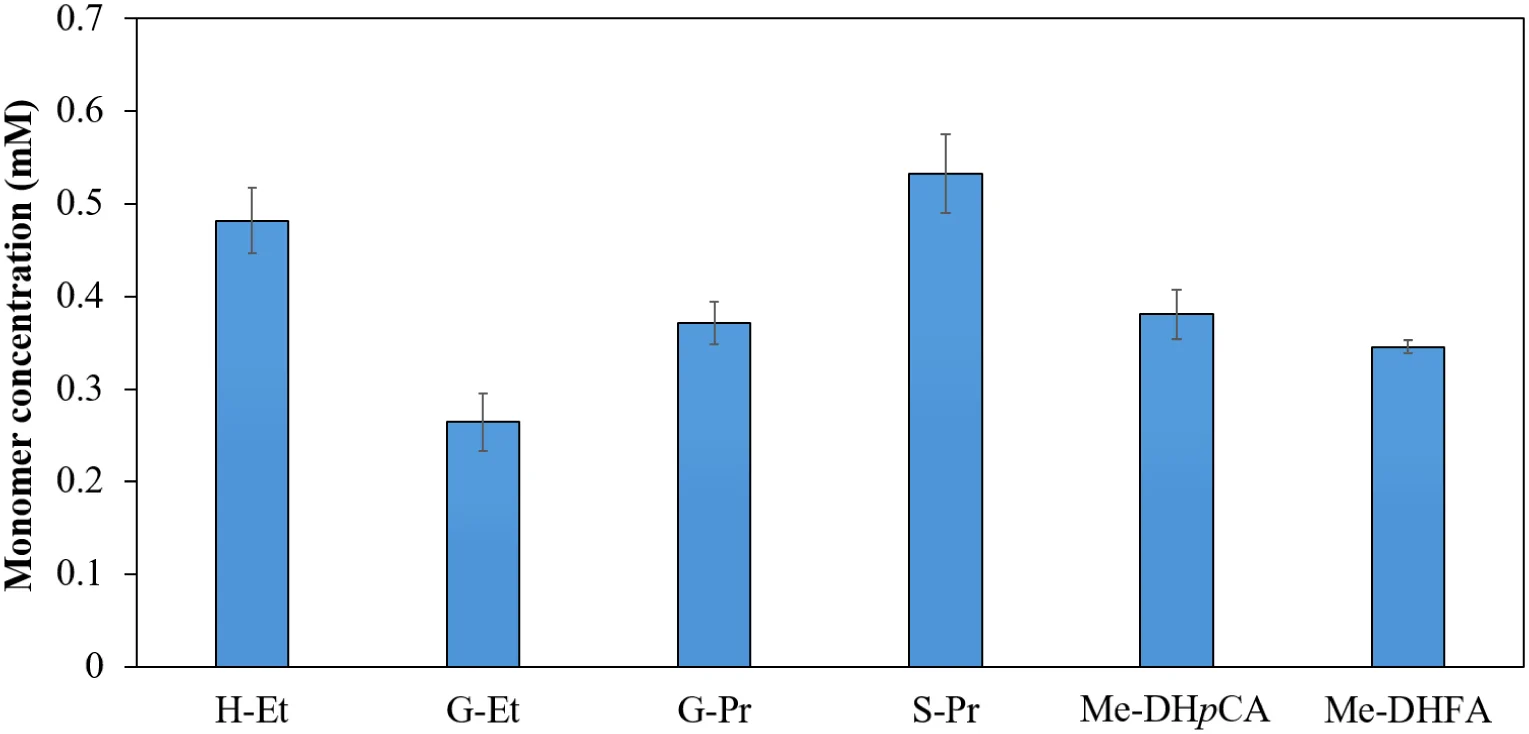
Monomer aromatics extracted from manure fibers by RCF. Abbreviations: H-Et: ethylphenol, G-Et: 4-ethyl-2-methoxyphenol, G-Pr: 4-propyl-2-methoxyphenol, S–Pr: 4-propyl-2,6-dimethoxyphenol, Me-DH*p*CA: methyl 7,8-dihydro-*p*-hydroxycoumarate, Me-DHFA: methyl 7,8-dihydroferulate. Error bars represent the standard deviation of triplicate samples.

The phenolic monomer yield from RCF was ∼14 g aromatics/kg manure fibers, which represents a 2.5-fold increase when compared to that obtained with the eAPL process (see Figure S3 for aromatic yields on a kg lignin basis). Since RCF deconstruction should provide access to the aromatic compounds that are in the lignin backbone as well as those present as pendant groups (e.g., *p*-coumaric acid), a higher yield was expected from RCF compared to the eAPL treatment that primarily targets the pendant groups. However, compared to aromatic yields from bioenergy crops, the yields from digested manure fibers were low.^18^

More importantly, RCF with the Pd/C catalyst preferentially produced ethyl and propyl phenolics (Figure 2) instead of the propanol phenolics that were reported to be produced when using the same catalyst and solvent in hydrogenolysis of lignin extracted from plant biomass.^18^ Perez et al.^18^ reported that for grasses (sorghum and switchgrass), only 18% of the extracted aromatics were ethyl and propyl phenolic compounds, whereas the majority of the extracted aromatics were methyl esters (56% in sorghum and 40% in switchgrass) and phenolic compounds with propanol side chains (23% in sorghum and 36% in switchgrass). They showed that these two major types of extracted aromatics (accounting for 79% of the extracted aromatics in sorghum and 76% in switchgrass) could be funneled to PDC.^18^ They also demonstrated that with the same *N. aromaticivorans* strain used in this study, the ethyl and propyl phenolic compounds were transformed to dead-end aryl ketones and not to PDC.^18^

The results with the digested manure fibers (Figure 2) showed that propanol phenolics were not extracted and that 33% of the extracted aromatics were methyl esters. With only the methyl esters being potentially transformed to PDC,^18^ this aromatic distribution predicts a low PDC yield from the RCF products obtained in this study. Using the methods of Perez et al.,^18^ we measured a PDC yield of 20 ± 5%, confirming the low-yield predictions.

Recently, Hall et al.^66^ reported that a single amino acid change on a previously uncharacterized gene (*marK*) was sufficient to enable *N. aromaticivorans* growth on acetovanillone and guaiacylpropanone. This is an important observation because Perez et al.^18^ demonstrated that with the *N. aromaticivorans* strain used in this study, the G-type ethyl phenolics were transformed to acetovanillone and G-type propyl phenolics were transformed to guaiacylpropanone and that these two aromatic ketones were dead-end products. Thus, in the future, it may be possible to construct *N. aromaticivorans* strains that are capable of converting both ethyl and propyl phenolics to PDC, using the genetic modification demonstrated by Hall et al.^66^ for G-type ethyl and propyl phenolics and other modifications for S-type ethyl and propyl phenolics.^67^ Such genetic engineering improvements could make the products of RCF from postdigestion manure fibers more compatible with microbial funneling to PDC.

The observed shift in the type of phenolics derived from the G and S units in lignin, from propanol phenolics to ethyl and propyl phenolics, could potentially be related to the presence of catalytic metals in the large fraction of ash present in the postdigestion fibers (Table 1). For instance, copper is used in the dairy industry to suppress infections of dairy cow hoofs,^68,69^ and if copper is abundant in the postanaerobic digestion fibers, it may contribute to catalytic activity occurring in the RCF process, resulting in a different monomeric product distribution than when using Pd/C with extracted lignin. Future investigations of the ash composition and its potential to catalyze hydrogenolysis may provide some answers to the unexpected monomeric product distribution.

### Biological Funneling of Manure-Derived Aromatics into PDC

Biological funneling of the extracted aromatics to PDC was further investigated with eAPL since the production of ethyl and propyl phenolics, instead of propanol phenolics, by the RCF process resulted in low PDC yields.

The procedure used for the preparation of eAPL aimed at reaching aromatic concentrations of ∼40 mM, which is desirable to produce PDC at high rates and with high titers.^16^ In addition, the eAPL preparation procedure seeks to reduce the concentration of Na^+^, which is typically high in other APLs^70,71^ but can inhibit growth of *N. aromaticivorans*.^16^ To prepare the feed solution for biological funneling experiments, the eAPL was amended with glucose (20 mM), phosphate buffer, and a vitamin solution (see Methods). Figure 3 shows the accumulation of PDC during a fed-batch experiment in which glucose serves as a growth substrate and the aromatics are converted to PDC. The cell density throughout the experiment remained stable. For pH control during PDC production, NH_4_OH was used instead of NaOH, since at high concentrations, NH_4_^+^ has been demonstrated to be less inhibitory to *N. aromaticivorans* than Na^+^.^16^ With a total concentration of aromatics in the feed solution being 30.6 mM, a total of 2.75 mmol of fiber-derived aromatics were added as feed and 2.71 mmol PDC were produced at the end of the experiment, resulting in a 98% yield on a molar basis. Further analysis of the data from this fed-batch reactor indicated a titer of 2.6 g PDC/L and a production rate of 0.2 g PDC/(L·h). The 98% molar yield indicated that the eAPL derived from manure fibers was biologically compatible with *N. aromaticivorans*, with no observed inhibitory effects throughout the experiment. This result demonstrates that aromatics extracted from the anaerobically digested manure fibers could indeed be used to produce the target chemical, and potentially other lignin-derived chemicals.^21,25^ Furthermore, the near-stoichiometric yield agrees with prior reports of PDC production from eAPL that was produced from poplar biomass.^16^

**Figure 3:**
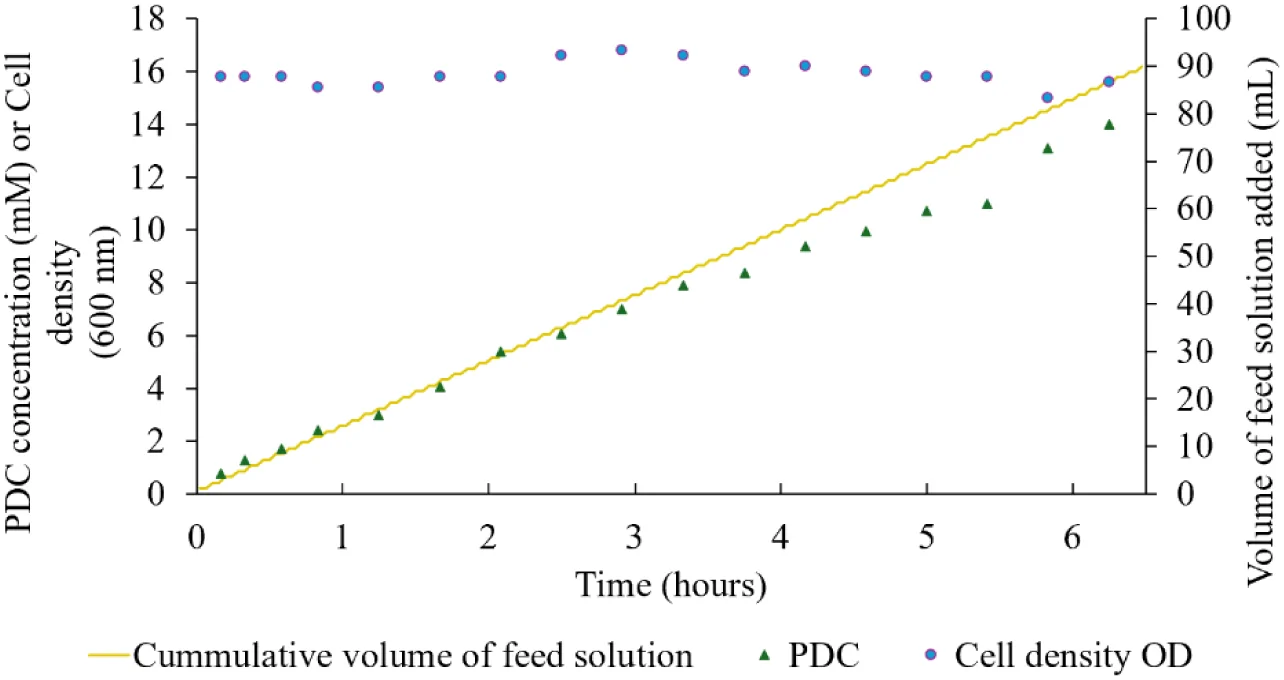
PDC production in fed-batch bioreactor experiment with glucose-amended eAPL as feed solution. The system was operated with the addition of 1 mL feed solution every 5 min. Cell density was measured throughout the experiment using absorbance at 600 nm.

Microbial production of PDC has been reported with different microbial catalysts using synthetic media containing aromatics as substrates,^72–75^ or aromatic-rich liquors from alkaline-pretreated biomass.^16–19^ In addition, processes for PDC purification from the biological funneling of aromatics have been reported.^16,74,76^ This study adds to this list the ability to produce PDC from aromatics extracted from postanaerobic digestion manure fibers (Figure 3, Table S2). The observed titer of 2.6 g PDC/L is modest in comparison to the highest reported titer of 99.9 g PDC/L when synthetic media was utilized^72^ or 9.2 g PDC/L from eAPL produced from poplar biomass.^16^ Likewise, the obtained production rate of 0.2 g PDC/(L·h) is modest compared to the 1.53 g PDC/(L·h) rate reported from poplar eAPL.^16^ A high PDC titer was also reported by Lee et al.^77^ who implemented oxidative lignin depolymerization of poplar and fed-batch bioreactor experiments with an engineered strain of *P. putida*, obtaining 12.3 g PDC/L at a production rate of 0.1 g PDC/(L·h). These comparisons suggest that there is room to improve rates and titers of PDC from digested manure fibers, if bioreactor optimization approaches are used, as reported with poplar biomass.^16^ Opportunities to increase titers, from aromatic liquors produced from post digestion fibers or other biomass, will depend on two factors: the development of extraction procedures that increase the aromatic concentrations in the liquors, and increasing the tolerance of PDC-producing strains to the concentrated liquors. Regarding the former, eAPL mostly targets the extraction of pendant groups in lignocellulosic biomass, and thus, solid residues from the eAPL process are expected to contain aromatic compounds that are potentially extractable and could increase the aromatic yield from the postdigestion fibers. Regarding liquor tolerance, subjecting *N. aromaticivorans* to adaptive laboratory evolution could result in strains better adapted to growing on the liquors produced from the digestate. Furthermore, Kim et al.^16^ showed that increasing PDC titers from 3.1 g PDC/L to 9.2 g PDC/L, and production rates from 0.13 g PDC/(L·h) to 1.53 g PDC/(L·h), with *N. aromaticivorans* funneling eAPL from poplar could be achieved by using high-density cultures and short retention times in flow-through bioreactors, suggesting that similar approaches could be used to increase titers and rates beyond the proof-of-concept baselines obtained in this study. The limits in the study of Kim et al. were associated with the accumulation of intermediate metabolites when the loading rates were too high.^16^

Technoeconomic analyses of PDC production from lignin-derived aromatics using *N. aromaticivorans* and RCF for lignin depolymerization have been reported.^18,57,78^ The most recently estimated minimum selling price (MSP) is $13.98 per kg of PDC.^57^ A technoeconomic analysis of PDC production by its accumulation *in planta* (e.g., in transgenic sorghum) has also been conducted, with the estimated MSP ranging from $4 to $36 per kg of PDC depending on the assumptions used.^79^ In the aforementioned study, a PDC-producing microbial system was also simulated, yielding an MSP of $4.8 per kg of PDC. These analyses yield a wide range of MSP predictions depending on the overall production pipeline and the assumptions used in the models, and they highlight that, in general, further process optimizations are needed to continue reducing the MSP of PDC, regardless of the selected process. Nevertheless, these analyses have identified key unit processes in PDC-producing pipelines that require modifications. For instance, an initial analysis with *N. aromaticivorans* and RCF identified biomass deconstruction and lignin depolymerization as critical steps that needed improvements.^18^ Upgrades in biomass fractionation reduced the estimated MSP by 29%.^78^

Although technoeconomic analyses are needed in the future to evaluate the production of PDC from digested manure fibers as the source of lignin, there are several reasons why these fibers could improve the cost analysis of a PDC production process compared with PDC production from other biomass sources. First, the digested fibers have a higher lignin content compared with other feedstocks, and therefore, a higher aromatic yield could be achieved per mass of feedstock treated. Second, the residual biomass present in the digested fibers has already undergone some level of deconstruction in the cow’s rumen and subsequently in the anaerobic digester. This may reduce the costs associated with biomass fractionation and lignin depolymerization. Third, the use of energy crops as a biomass source is generally subject to seasonal availability, as harvesting is limited to specific periods during the year. In contrast, cow manure is produced daily, and anaerobic digesters have a constant and steady supply of solid digestate, which may bring additional benefits to a biomass-to-PDC production pipeline.

Finally, the potential variability in the composition of digested manure fibers (Table 1), which is expected to be a function of animal diet or anaerobic digester operational parameters, may influence the effectiveness of different processes in a PDC-producing pipeline, thereby requiring further investigation now that the feasibility of using digested manure fibers as a source of lignin has been demonstrated in this study.

## Conclusions

In this study, it was demonstrated that lignin aromatics remain after anaerobic digestion of dairy manure and that these can be extracted and used for the production of a value-added product such as PDC. This investigation adds to the potential uses of dairy manure, an abundant yet underutilized, lignocellulosic biomass resource. Moreover, by focusing on postanaerobic digestion manure fibers, the study suggests that it is possible to initially extract biogas from manure, while recovering the nutrient-rich liquid fraction of the digestate to be used as fertilizer, and then obtain additional value from the aromatics present in the digested manure fibers by using them for microbial production of organic compounds that would reduce reliance on fossil-fuel sources.

## Experimental Methods

### Source and Storage of Postanaerobic Digestion Manure Fibers

Samples of postanaerobic digestion manure fibers were collected in 5-gallon buckets from a fiber pile generated after separation of liquid and solid digestate, in a mesophilic plug-flow-type anaerobic digester at Fair Oaks (IN, USA) that processes manure from a 12,000-cow dairy farm. The moisture content of the fibers, as received, was 57% ± 1%. A portion of these fibers was stored at 4 °C to minimize microbial activity, whereas another portion was dried in an oven (Fischerbrand Isotemp oven, Fischer Scientific, PA, USA) at 80 °C for 20 h to obtain a moisture content of ∼5%, which facilitated storage in sealed plastic bags at room temperature. From these sources, between 400 and 500 g of material was retrieved at different times to provide starting material for the various experiments performed.

### Compositional Analysis

Dried samples of anaerobically digested manure fibers were submitted to Dairyland Laboratories (Arcadia, WI, USA) for compositional analysis using standardized methods^80^ that included neutral detergent fiber (NDF) using AOAC Official Method 2002.04, acid detergent fiber (ADF) and lignin using AOAC Official Method 973.18; ash with AOAC Official Method 942.05, and crude protein with AOAC Official Method 990.03. For each sample, these analyses were performed in triplicate. Lignin, ash, and crude protein contents were provided in the laboratory reports. The hemicellulose content was calculated by subtracting ADF from NDF (i.e., %NDF - % ADF),^34^ and cellulose content was obtained by subtracting lignin content from ADF (i.e., %ADF - %lignin).^34^

### NMR Analyses

The lignin content of the postanaerobic digestion manure fibers was analyzed by NMR using standard protocols.^81^ Briefly, a fraction of the dried fibers (1 g) was ball-milled for 13 h in 20 mL agate jars with 10 mm-diameter agate milling balls using a Fritsch Pulverisette 7 planetary ball mill (Fritsch, NC, USA) operating at 600 rpm for 50 cycles of 10 min grinding, followed by 5 min breaks to avoid excessive heating. Subsequently, to remove most of the structural carbohydrates, the ball-milled samples (900 mg) were suspended in 45 mL of 25.5 mM sodium acetate buffer (pH 5.0) and then treated with Cellulysin cellulase (from *Trichoderma viride*, Calbiochem, MilliporeSigma, Burlington, MA, USA) at a ratio of 1:10 (mass/mass) of enzyme to ball-milled fibers, respectively. The samples were incubated in a shaker (New Brunswick Innova 2300, Eppendorf SE, Hamburg, Germany) at 250 rpm for 2 days at 35 °C. After the first digestion was completed, the samples were collected via centrifugation at 8000 rpm for 10 min on a Sorvall Biofuge centrifuge (ThermoFischer, Waltham, MA, USA). The supernatant was decanted, and the 2-day enzymatic digestion process was repeated on the solids. After two enzymatic digestion cycles, the samples were washed three times with reverse osmosis water by suspending the solids in the water, pelletizing, and decanting the water. Finally, the samples were frozen at −20 °C and subsequently lyophilized (pressure <20 mTorr) to produce an enzyme-treated lignin material.^81^

NMR experiments were performed with ∼20 mg of enzyme-treated lignin material dissolved in 500 μL of 4:1 DMSO-*d*_6_/pyridine-d_5_ (v/v). Heteronuclear single quantum coherence (HSQC) spectra were collected on a cryoprobe-equipped NEO 700 MHz spectrometer (Bruker Corp., Billerica, MA, USA) with an adiabatic-pulse program (hsqcetgpsisp2.2). HSQC experiments were carried out using the following parameters: acquired from 11.65 to −0.66 ppm in F2 (^1^H) with 3,448 data points (acquisition time, 200 ms) and 215 to −5 ppm in F1 (^13^C) with 618 increments (F1 acquisition time, 8 ms) of 24 scans with a 1 s interscan delay; the d_24_ delay was set to 0.89 ms (1/8J, J = 140 Hz). The total acquisition time for a sample was 6 h. Processing used typical matched Gaussian apodization (GB = 0.001, LB = −0.5) in F2 and a squared cosine-bell in F1 (without using linear prediction). The spectra were referenced using the central DMSO solvent peak (δ_C_ 39.5, δ_H_ 2.49 ppm). Peak identifications were based on previous reports.^82,83^ The volume integration of contours in HSQC plots was performed manually using fixed rectangular integration boxes in the TopSpin 4.1.4 software, and no correction factors were used; that is, the data represent volume integrals only. The relative monolignol composition (percentage) was determined from fixed integration regions and expressed on the basis where 100% of aromatics = G_2_ + 1/2 S_2/6_; % *p*CA = 1/2 *p*CA_2/6_ / ((Methoxy * % S/6) + (Methoxy * %G/3)); A = A_α_ / ((Methoxy * %S/6) + (Methoxy * %G/3)).^81^

### Aromatic Extraction and Lignin Depolymerization from Postanaerobic Digestion Manure Fibers

Two methods were used to extract aromatics from the postanaerobic digestion manure fibers. One method consisted of alkaline pretreatment followed by acidic digestion and liquid–liquid extraction^16^ to generate the eAPL solution. The second method is RCF, which deconstructs the biomass and depolymerizes lignin.^18^

To produce the eAPL, 69 g of the digested manure fibers as received were mixed with 160 mL of 1 M NaOH in a 500 mL Pyrex screw-cap glass bottle, and then digested at 121 °C for 1 h at 1.04 bar.^16^ This process was performed in five separate bottles, for a total of 345 g of biomass and 800 mL of 1 M NaOH used in the process. The alkaline treatment was followed by an acid hydrolysis step performed by adding 14.4 mL of 32% H_2_SO_4_ to each bottle to adjust the pH to ∼2, and the mixture was subsequently digested at 121 °C for 1 h at 1.04 bar. The hydrolyzed biomass collected from all five bottles was mixed with 500 mL deionized (DI) water and 1 L of 100% ethyl acetate, and then, the mixture was vacuum-filtered with a cellulose Whatman Grade 2 filter (185 mm diameter with 8 μm pore size). Next, a liquid–liquid separation of the water and ethyl acetate was performed in a separation funnel. The aqueous fraction was washed with 200 mL of ethyl acetate before being discarded, and the organic fractions containing the phenolic compounds were combined and collected. The solid pulp was subjected to a second filtration after adding 1 L of ethyl acetate and 1 L of DI water. Subsequently, the aqueous and organic fractions were allowed to separate in the separation funnel. A total of ∼1.6 L of aromatic-rich ethyl acetate solution was pooled together in a 2 L round-bottom flask. The ethyl acetate was then removed using a rotary evaporator (Heidolph Hei-VAP Value, Wood Dale, IL, USA) operated at 60 °C. The extracted phenolics remained as a concentrated viscous material in the flask and were redissolved using 100 mL of 0.2 M NH_4_OH to produce the eAPL solution.

For RCF, dried postanaerobic digestion manure fibers were ground to a fine flour with a Retsch MM-400 shaker mill (Verder Scientific, Newtown, PA, USA) and subjected to RCF using hydrogen over palladium on carbon (5 wt % Pd/C) catalyst in methanol in a 50 mL Hastelloy Parr reactor.^18^ For the reaction, 1 g of ground fibers, 100 mg Pd/C, 30 mL methanol, and 0.22 mg/mL 1,2-dimethoxybenzene (internal standard) were added. The reactor was purged with argon and pressurized with hydrogen gas to 30 bar, then heated to 200 °C and held at this temperature for 4 h. The reactor was then rapidly cooled back to room temperature and slowly depressurized. The catalyst and residual solids were removed by a 1 μm PTFE (polytetrafluoroethylene) filter, and the resulting mixture of phenolics in 30 mL of methanol was stored at 4 °C for further analysis.

### Bioconversion of Fiber-Derived Phenolics into PDC

Experiments were performed using a 250 mL bench-top bioreactor (Infors HT–Multifors® 2, Bottmingen, Switzerland) controlled by the bioprocess software eve® (Infors HT, Bottmingen, Switzerland). The reactor was operated in a fed-batch mode with automated control of stirring speed, pH, air supply, and temperature. The feed solution was prepared by amending 90 mL of eAPL with 3.6 mL of 0.5 M glucose, 3.6 mL of 0.5 M Na_2_HPO_4_, 3.6 mL of 0.5 M KH_2_PO_4_, and 1.8 mL of Hutner’s vitamin-free concentrated base.^16^ The bioreactor was initially inoculated with 5 mL of an overnight-grown culture of a PDC-producing *N. aromaticivorans* DSM12444 strain (i.e., with deletion of *ligI* and *desCD* genes as described elsewhere^17^) and supplemented with 100 mL of standard mineral base (SMB) medium containing 50 mM glucose to promote initial growth overnight.^16^ Then, the system received 1 mL of feed solution every 5 min. The incubations were performed at 30 °C, with an air supply set at 0.4 L/min, stirring speed at 800 rpm, and pH 7, which was controlled with acid and base solutions containing 0.5 M HCl and 0.5 M NH_4_OH, respectively. The reactor was operated in fed-batch mode for 6.2 h. The initial volume of culture after pre-growing the cells was 100 mL, and a total of 90 mL of feed solution was pumped into the reactor, for a final volume of 190 mL at the end of the experiment. Cell growth was monitored with optical density measurements at 600 nm (OD_600_). Samples collected from the reactor for other analyses were centrifuged for 5 min at 5,000 rpm, and the supernatant was filtered through 0.2 μm PES filters. The filtrate was stored at −80 °C for preservation until further analyses.

Experiments were also performed with phenolics from RCF extraction, following procedures described by Perez et al.,^18^ in which cells were grown in a mixture of 9.8 mL of SMB medium containing 10 mM glucose and 0.2 mL of a methanol solution containing the products of lignin depolymerization. After 48 h of incubation, the cultures were centrifuged at 5,000 rpm for 5 min, and the resulting supernatants were used to measure the concentration of PDC produced.

### Quantification of Extracted Aromatic Compounds and PDC

Identification and quantification of aromatics in eAPL and of PDC from bioconversion experiments were performed on a liquid chromatography triple-quadrupole mass spectrometer (LC-TQMS) (Nexera XR HPLC-8045 MS/MS, Shimadzu, MD, USA). The mobile phase consisted of a dual gradient of solvent A (0.1% formic acid in DI water) and solvent B (0.1% formic acid in a 2:1 mixture of acetonitrile and methanol, v/v) as described elsewhere.^17^ The stationary phase was a Kinetex F5 column (2.6 μm pore size, 2.1 mm ID, 150 mm length column, Phenomenex, Torrance, CA, USA). Quantitative analysis of aromatic monomers in RCF was performed on a Shimadzu GCMS-TQ8030 equipped with an AOC-20i autosampler and an Rxi-5Sil MS column (15 m × 0.25 mm × 0.5 μm), helium gas mobile phase held at a constant linear velocity of 45 cm s^–1^, and the injection port was set to 250 °C with a split ratio of 20:1. The temperature program was as follows: 50 °C for 1 min, then ramped to 300 °C at 20 °C min^–1^, and held at 305 °C for 16.5 min.

## Supplementary Material



## Notes

Compositional analysis of digester manure fibers, PDC yields compared with other lignocellulosic feedstocks, and yields of aromatics after eAPL and RCF treatments (PDF)
