# Supplementary material for: Postanaerobic Digestion Manure Fibers as a Renewable Source of Lignin-Derived Aromatic Compounds for Microbial Upgrading
Source: ACS ES T Eng. 2026 Sep 9;6(10):2922–32. doi: 10.1021/acsestengg.6c00440 (PMC13660397; doi:10.1021/acsestengg.6c00440)
Supplement: Supplementary file 1 [file ee6c00440_si_001.pdf]

Supplementary Material for:

Post-anaerobic digestion manure fibers as a renewable source of lignin-derived aromatic compounds for microbial upgrading

Brayan D. Riascos, Bumkyu Kim, Steven D. Karlen, Jason Coplien, Dennis V. Haak, Sarada Sripada, Canan Sener, Timothy J. Donohue, K.G. Karthikeyan, Daniel R. Noguera

Table S1. Compositional analysis of digested manure fibers

	Biomass composition (% dry matter)						
**Sample**	**NDF***	**ADF***	**Lignin***	**Cellulose****	**Hemicellulose****	**Crude protein***	**Ash***
1.1	51.67	49.66	29.15	20.51	2.01	21.45	22.35
1.2	56.04	52.20	30.78	21.42	3.84	21.85	22.73
1.3	52.22	40.72	29.20	11.52	11.50	21.52	22.63
Average Sample 1	53.31	47.53	29.71	17.82	5.78	21.61	22.57
2.1	52.36	50.54	31.39	19.15	1.82	21.99	23.46
2.2	50.42	49.41	31.10	18.31	1.01	22.12	23.10
2.3	50.88	44.74	32.25	12.49	6.14	22.07	22.70
Average Sample 2	51.22	48.23	31.58	16.65	2.99	22.06	23.09
3.1	50.29	49.32	28.82	20.50	0.97	21.55	21.93
3.2	54.83	49.71	34.88	14.83	5.12	22.16	22.90
3.3	54.85	49.82	30.08	19.74	5.03	21.66	21.87
Average Sample 3	53.32	49.62	31.26	18.36	3.71	21.79	22.23
**Average**	**52.62**	**48.46**	**30.85**	**17.61**	**4.16**	**21.82**	**22.63**
**Standard deviation**	**0.99**	**0.87**	**0.82**	**0.71**	**1.18**	**0.19**	**0.35**

*Measured parameters: NDF = neutral detergent fiber; ADF = acid detergent fiber
**Calculated parameters: Cellulose = ADF – Lignin; Hemicellulose = NDF - ADF

Table S2. Comparison of lignin content, aromatic yields, and PDC yields using post-anaerobic digestion manure fibers and other lignocellulosic feedstocks reported in the literature

Lignocellulosic feedstock	Lignin content (%w/w)	Aromatic extraction method	Aromatic yield (g aromatics/ kg biomass)	PDC yield (g PDC/kg biomass)	Reference
Post digestion fibers	30	eAPL	5.6	1.6	This study
Poplar	24[a]	APL	2.3	2.7	Umana et al. (2022)[1]
Sorghum	16[a]	APL	8.8	9.3	Umana et al. (2022)[1]
Corn stover	14.3	APL-GVL[b]	8.9	-	Timokhin et al. (2020)[2]

a: Perez et al. 2022[3]
b: γ-valerolactone treatment

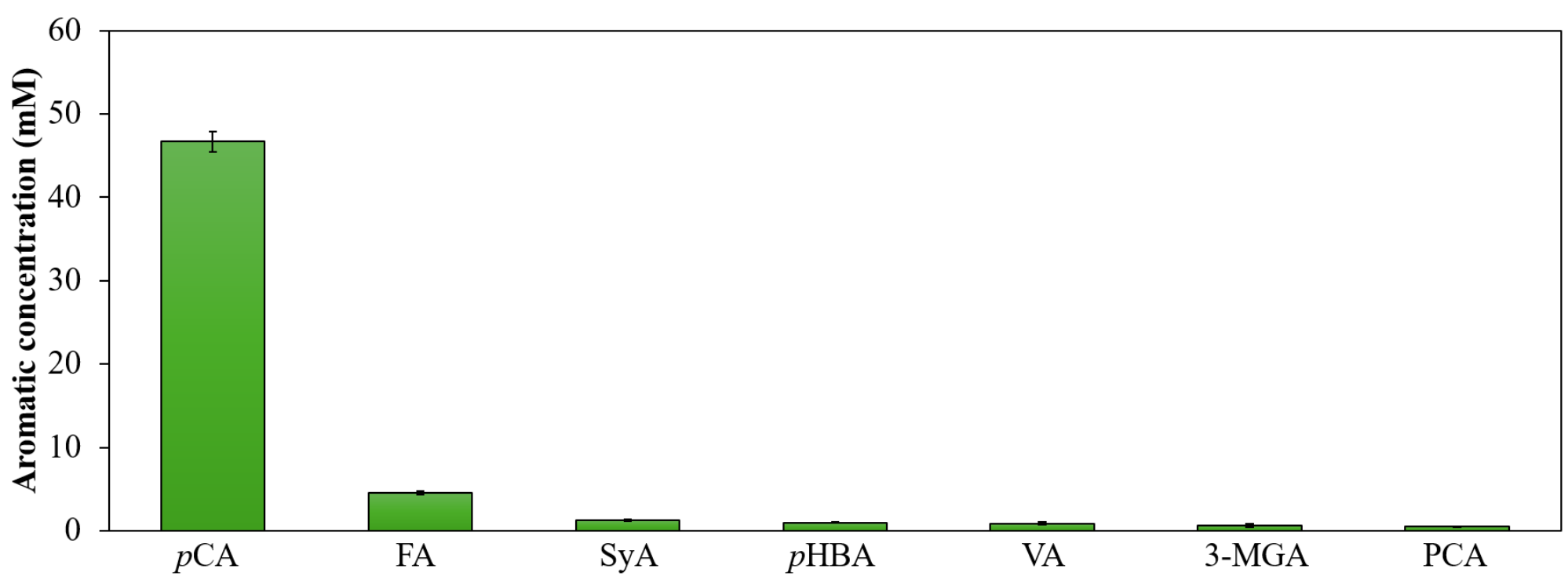

Figure S1. Aromatics in concentrated eAPL from post-anaerobic digestion manure fibers. Abbreviations: *p*CA: *p*-coumaric acid, FA: ferulic acid, SyA: syringic acid, *p*HBA: *p*-hydroxybenzoic acid, VA: vanillic acid, 3-MGA: 3-methylgallic acid, PCA: protocatechuic acid. Error bars represent the standard deviation of triplicate samples.

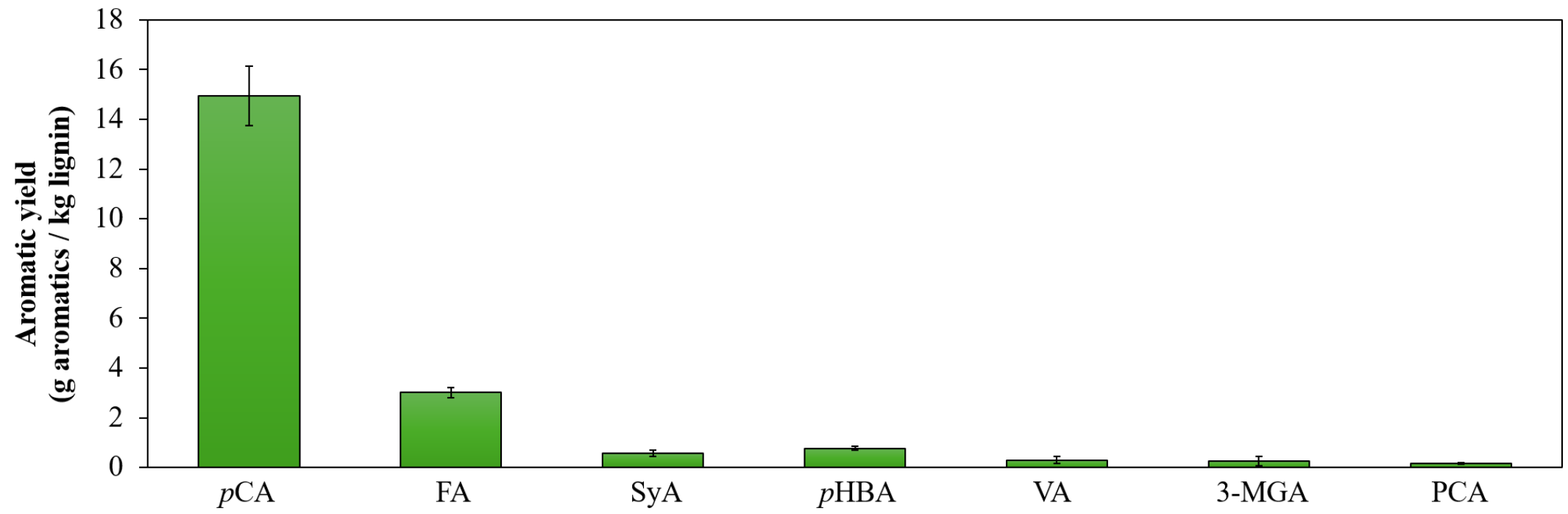

Figure S2. Yield of aromatics (g aromatic per kg lignin) in concentrated eAPL produced from post-anaerobic digestion manure fibers. Abbreviations: *p*CA: *p*-coumaric acid, FA: ferulic acid, SyA: syringic acid, *p*HBA: *p*-hydroxybenzoic acid, VA: vanillic acid, 3-MGA: 3-methylgallic acid, PCA: protocatechuic acid. Error bars represent the standard deviation of triplicate samples.

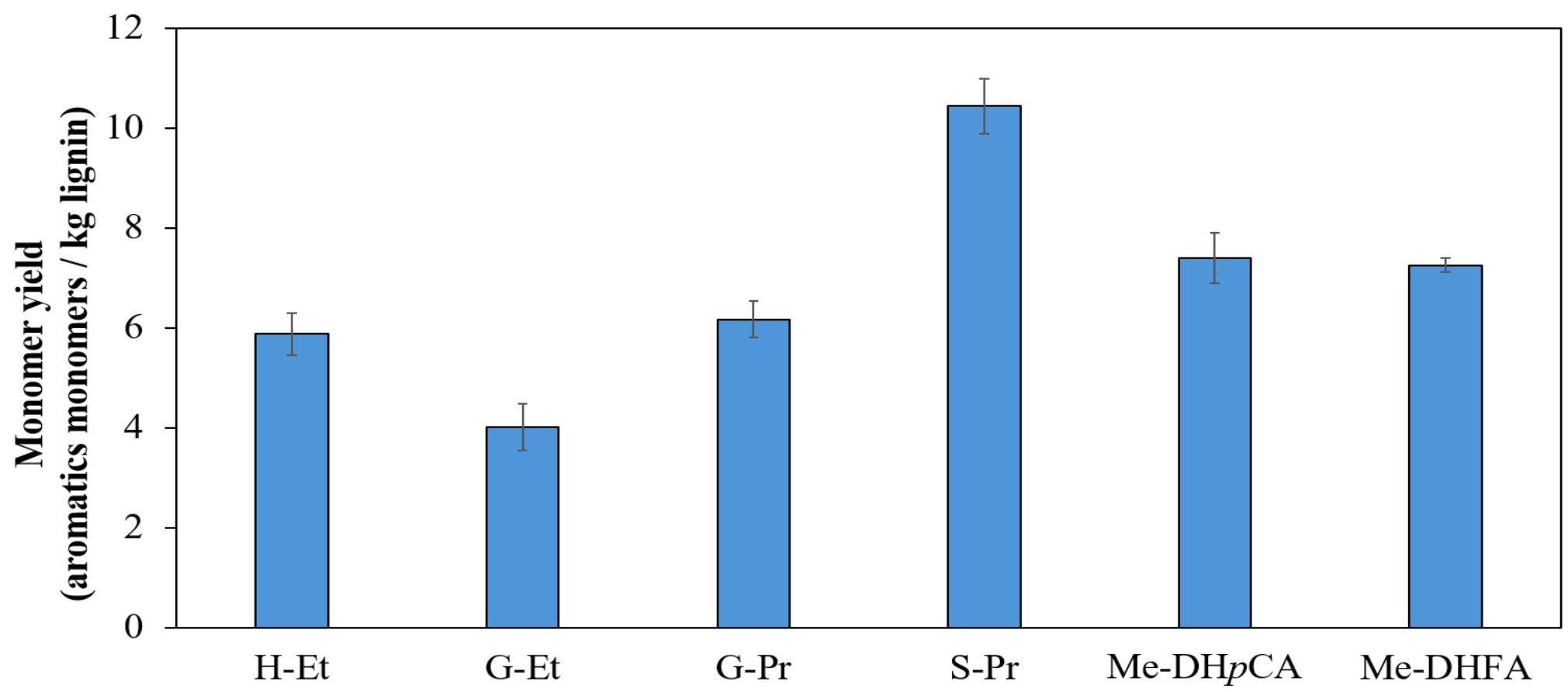

Figure S3. Yield of aromatics (g aromatic per kg lignin) extracted from post-digestion manure fibers by RCF. Abbreviations: H-Et: ethylphenol, G-Et: 4-ethyl-2-methoxyphenol, G-Pr: 4-propyl-2-methoxyphenol, S-Pr: 4-propyl-2,6-dimethoxyphenol, Me-DH*p*CA: methyl 7,8-dihydro-*p*-hydroxycoumarate, Me-DHFA: methyl 7,8-dihydro-ferulate. Error bars represent the standard deviation of triplicate samples.

References

(1) Umana, G. E.; Perez, J. M.; Unda, F.; Lin, C.-Y.; Sener, C.; Karlen, S. D.; Mansfield, S. D.; Eudes, A.; Ralph, J.; Donohue, T. J.; et al. Biological funneling of phenolics from transgenic plants engineered to express the bacterial 3-dehydroshikimate dehydratase (*qsuB*) gene. *Frontiers in Chemical Engineering* 2022, 4. DOI: 10.3389/fceng.2022.1036084.

(2) Timokhin, V. I.; Regner, M.; Motagamwala, A. H.; Sener, C.; Karlen, S. D.; Dumesic, J. A.; Ralph, J. Production of *p*-coumaric acid from corn GVL-lignin. ACS Sustainable Chemistry & Engineering 2020, 8 (47), 17427-17438. DOI: 10.1021/acssuschemeng.0c05651.

(3) Perez, J. M.; Sener, C.; Misra, S.; Umana, G. E.; Coplien, J.; Haak, D.; Li, Y.; Maravelias, C. T.; Karlen, S. D.; Ralph, J.; et al. Integrating lignin depolymerization with microbial funneling processes using agronomically relevant feedstocks. *Green Chemistry* 2022, 24 (7), 2795-2811. DOI: 10.1039/d1gc03592d.
